# Experimental and Theoretical Study of Ultra-Hard AlMgB_14_-TiB_2_ Composites: Structure, Hardness and Self-Lubricity

**DOI:** 10.3390/ma15238450

**Published:** 2022-11-27

**Authors:** Pavel Nikitin, Ilya Zhukov, Dmitrii Tkachev, Yurii Abzaev, Ekaterina Marchenko, Alexander Vorozhtsov

**Affiliations:** 1 Laboratory of Metallurgy Nanotechnologies, National Research Tomsk State University, Lenin Avenue, 36, 634050 Tomsk, Russia; 2 Department of Higher Mathematics, Tomsk State University of Architecture and Building, Solyanaya Sq., 2, Building 2, 634003 Tomsk, Russia; 3 Laboratory of Superelastic Biointerfaces, National Research Tomsk State University, Lenin Avenue, 36, 634050 Tomsk, Russia

**Keywords:** ceramics, DFT, tribo-layer, friction, tribology, nanohardness

## Abstract

It is known that the presence of oxygen phases in hard materials leads to an undesirable decrease in the mechanical properties. In materials based on AlMgB_14_, the main oxygen impurity is spinel MgAl_2_O_4_; it significantly reduces the hardness of AlMgB_14_ and its formation during sintering is inevitable. In this work, the ultra-hard spark plasma sintered (SPSed) AlMgB_14_-TiB_2_ composite material was fabricated from the AlMgB_14_-TiB_2_ precursor obtained by self-propagating high-temperature synthesis (SHS). Due to the high synthesis temperatures, the main oxygen phase in the obtained composite was Al_4_B_2_O_9_ instead of spinel MgAl_2_O_4_. It was found that the obtained composite has excellent mechanical properties. The maximum hardness of the sample is 44.1 GPa. The presence of oxygen in the form of the Al_4_B_2_O_9_ phase led to unexpected results: the friction coefficient of the obtained AlMgB_14_-TiB_2_ composite under dry conditions against the Al_2_O_3_ counter-specimen is approximately four times lower than the friction coefficient of pure ceramic AlMgB_14_ (0.18 against 0.7, respectively). Based on the observed results, it was found that the Al_4_B_2_O_9_ particles formed during the SHS are responsible for the low friction coefficient. The quantum chemical calculations showed that the elastic moduli of Al_4_B_2_O_9_ are significantly smaller than the elastic moduli of AlMgB_14_ and TiB_2_. Thus, during sliding, Al_4_B_2_O_9_ particles are squeezed out onto the composite surface, form the lubricating layer and reduce the friction coefficient.

## 1. Introduction

Currently, AlMgB_14_-based materials (BAMs) are of great interest due to their unique physical and mechanical properties. BAMs have an extremely low friction coefficient (COF, ~0.08–0.02) [1,2,3], a high hardness, with value reaching 32 GPa [4], a coefficient of thermal expansion (CTE) close to steel (11.7 × 10^−6^ K^−1^) [5], and a high thermal and chemical stability. Although AlMgB_14_ single crystals were first obtained in 1970 by Matkovich and Economy [6], polycrystalline materials based on AlMgB_14_ have been actively studied since 2000 in the Ames Laboratory (USA) [2,3,4]. First, the scientists of [4] found that the addition of TiB_2_ increases the hardness of the AlMgB_14_-TiB_2_ composite to 40–46 GPa, and then the studies of the tribological characteristics showed that the friction coefficient of the AlMgB_14_ coating in dry conditions can reach 0.05 [3], while the friction coefficient of the AlMgB_14_-TiB_2_ coating in a water-glycol lubricant can reach values of 0.02 [1]. Due to the unique combination of these properties, AlMgB_14_ has attracted great attention as a wear-resistant material in the friction units of critical machine parts.

Tribological analysis showed that the extremely low friction coefficient in dry conditions and water-glycol lubricants is associated with the release of B_2_O_3_ and B(OH)_3_ particles [1,2,3] during the friction. Studies of the tribological characteristics of AlMgB_14_-TiB_2_ composites in dry conditions [7] revealed that the COF of AlMgB_14_-30 wt% TiB_2_ at room temperature varies in the range from 0.45 to 0.65 against the SiC counterface and decreases significantly to 0.2 at 800 °C. The authors of [7] attribute this effect to the formation of TiO_2_ and B_2_O_3_ oxygen compounds on the contact surfaces of materials. Studies of the tribological characteristics of AlMgB_14_-Si composites [8] have shown that the friction coefficient under dry conditions varies from 0.19 to 0.28 when sliding against the 316 L counterface. A low COF is associated with the release of Si particles onto the worn surface. A similar effect was observed in [9]: studies have shown that one of the reasons for the low friction coefficient (COF, 0.12) of Cu/AlMgB_14_ composites is that AlMgB_14_ particles are squeezed out of a rigid Cu matrix onto a worn surface under the action of external stress.

Based on the given data, it can be concluded that the low friction coefficient of various composites AlMgB_14_-X (X = TiB_2_, Si, Cu) corresponds to the release of certain particles on the surface of the material during friction. These particles lubricate the surface, reducing the COF and wear, respectively. They are released either in the rigid metal matrix [8,9], or when using certain lubricants [1,2,3], or when the surface is heated [7]. At the same time, the question of the mechanism of the self-lubrication of bulk composites AlMgB_14_-TiB_2_ in dry conditions at room temperature remains open.

In this work, the AlMgB_14_-TiB_2_ precursor was produced using a cost-effective technology of self-propagating high-temperature synthesis (SHS) [10]. Thereafter, the bulk AlMgB_14_-TiB_2_ composite was fabricated using the spark plasma sintering (SPS) method. The mechanism of the self-lubricity, phase composition, structure and properties of the obtained AlMgB_14_-TiB_2_ composite were studied.

## 2. Materials and Methods

### 2.1. Materials Preparation

To obtain the AlMgB_14_-TiB_2_ precursor, a simple and low-cost technology of SHS was used. (Ti + 2B) exothermic mixture was chosen as the donor component. For this, 69 wt% of titanium was mixed with 31 wt% of boron. The mixture of an intermetallic alloy Al_12_Mg_17_ (the process of obtaining the Al_12_Mg_17_ powder is described in detail in [11] and amorphous boron powder) was used as an acceptor mixture. For this, Al_12_Mg_17_ powder was mixed with B powder in the ratio of 25 wt% Al_12_Mg_17_–75 wt% B. The characteristics of the raw precursors are given in Table 1. The obtained powders were mixed in the ratio of 50 wt% (Al_12_Mg_17_: B)–50 wt% (Ti + 2B). The mixing was carried out in ethanol. After drying in a vacuum furnace at 200 °C for 4 h, a sample was prepared from the obtained mixture, which was then synthesized by the SHS method. The process of obtaining AlMgB_14_-TiB_2_ precursors by SHS is described in detail in [10].

In the second set, the produced AlMgB_14_-TiB_2_ precursor was consolidated and sintered by spark plasma sintering (DR. SINTER model SPS-625 Spark Plasma Sintering System, SPS SYNTEX INC. Ltd., Tokyo, Japan) in a 12.8 mm graphite die in an argon atmosphere. The sintering temperature was 1500 °C (the heating rate was 50 °C/min without holding time). The pressure was 70 MPa. The diameter and thickness of the obtained composite were ~12.5 mm and 3 mm, respectively. After sintering, the sample was taken out from the die, and the surface of the sample was polished with diamond pastes (the maximum surface roughness was 0.5 μm).

### 2.2. Measurements

The combustion temperature was measured using a tungsten-rhenium thermocouple (Promelectronica, Yekaterinburg, Russia). The morphology, elemental composition and grain size of the AlMgB_14_-TiB_2_ precursor were observed by scanning electron microscopy (JEOL JSM-6490, JEOL, Tokyo, Japan) at BEC (backscattered electron) and MIX (backscattered electron + secondary electron) modes equipped with an energy dispersive spectroscopy (EDX). To determine the amount of oxygen in the initial powders, a LECO ONH (St. Joseph, MI, USA) analyser was used. X-ray structural studies of the AlMgB_14_-TiB_2_ composite material were carried out on a Shimadzu 7000 (Kyoto, Japan) diffractometer. The phase composition was refined using the Rietveld method with the Diffrac.EVA program and Powder Diffraction File database. The theoretical XRD pattern was obtained by combining three reference XRD patterns of AlMgB_14_, TiB_2_ and Al_4_B_2_O_9_ phases. The CASTEP program code [12,13] was used to calculate the free energies (E) of the reference and refined crystal lattices within the framework of the density functional theory (DFT) using the generalized-gradient approximation (GGA). Ultrasoft pseudopotentials were used, and the cutoff energy was 500 eV. The density of the AlMgB_14_-TiB_2_ composite was calculated by Archimedes’ principle in distilled water. The nanohardness was measured using a benchtop nanoindentation system (CSM Instruments— Peseux, Switzerland). The load was 300 mN, and the holding time was 10 s. The determination of the nanohardness was carried out by the methods of Oliver and Pharr [14]. The Berkovich indenter was used in the work. The obtained nanohardness results were converted into Vickers microhardness. The friction coefficient was measured on a pin-on-disk tribometer (TRIBOTester, TRIBOtechnic, San Francisco, CA, USA). Al_2_O_3_ ball was used as a counter-specimen. The normal load was 5 N, and the speed was 25 mm/s with the test duration of 1500 s. The tests were performed under normal conditions at room temperature in air without using a lubricant coating. The topographic and energy dispersive (EDX) analysis of the near-surface regions of the sample after the sliding friction was performed on a Tescan Mira 3 (TESCAN, Brno, Czech Republic) scanning microscope.

## 3. Results

### 3.1. Combustion Temperature and Morphology of the AlMgB_14_-TiB_2_ Precursor

**Figure 1:**
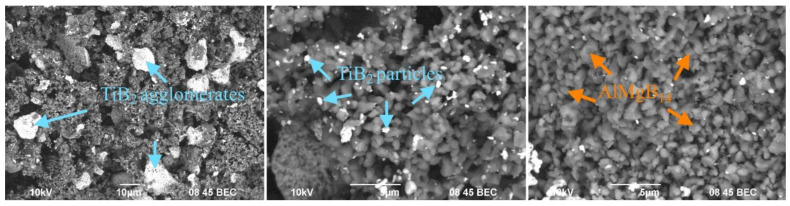
SEM images of the AlMgB_14_-TiB_2_ precursor (backscattered electron).

The results of measuring the temperature (the heat pattern is given in the Supplementary Materials) showed that during the exothermic reaction (Ti + 2B), the temperature increases to 1980 °C, and titanium diboride grains are formed. The heat released during the reaction is spent on the reaction in the Al_12_Mg_17_:B acceptor mixture with the formation of AlMgB_14_ [15]. According to the presented SEM images (Figure 1), the produced AlMgB_14_-TiB_2_ precursor consists of particles with an average size of 1 μm. EDX analysis (given in Supplementary Materials) showed that Ti and B elements were found in the light areas, which corresponds to the TiB_2_ phase. The B, Al and Mg elements were found in the dark areas, which corresponds to the AlMgB_14_ phase. The titanium diboride particles (light particles) also form agglomerates. The formation of TiB_2_ agglomerates is due to the fact that the heat released during the reaction (Ti + 2B) also leads to the fusion of TiB_2_ grains [16,17].

### 3.2. Phase Composition of the AlMgB_14_-TiB_2_ Composite

**Figure 2:**
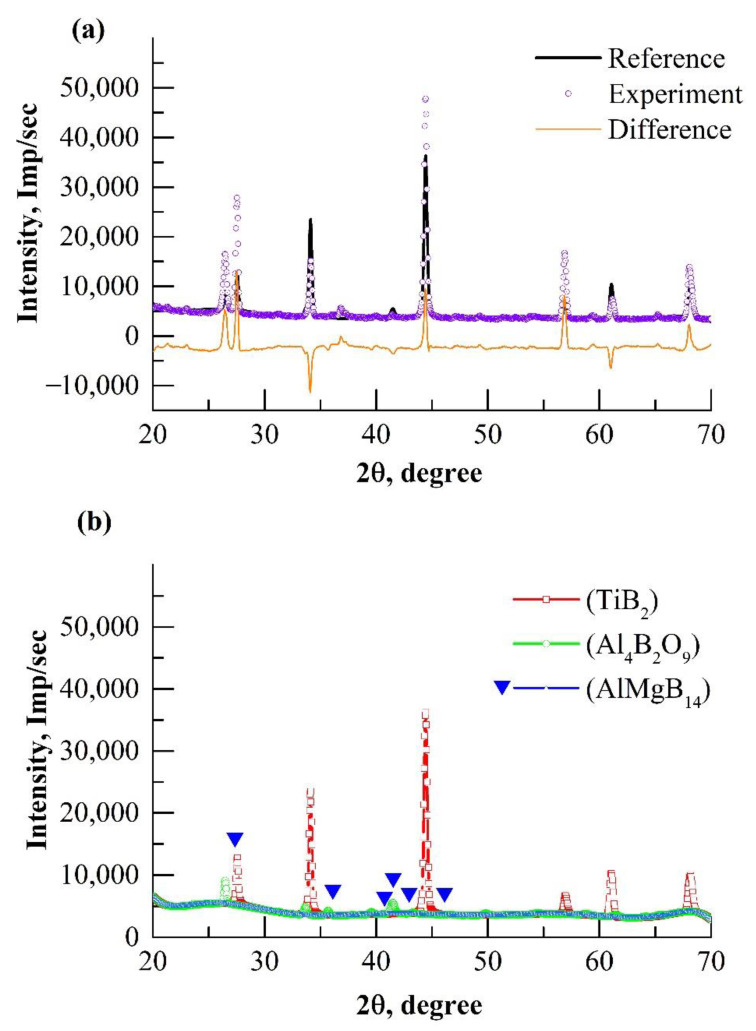
XRD patterns of the spark plasma sintered AlMgB_14_-TiB_2_ composite: (**a**) reference and experimental XRD patterns; (**b**) XRD patterns of the phases, found by the Rietveld method.

The results of the XRD analysis of the spark plasma sintered AlMgB_14_-TiB_2_ composite are shown in Figure 2. The results of the X-ray structural analysis are shown in Table 2 (a, b, c, α, β, γ are the structural parameters, V is the volume of the crystal lattices and E is the free energy of crystal lattices of the detected phases). The analysis of the contributions to the integral intensity of the individual phases (Table 2, Figure 2) showed that the main phases are TiB_2_, AlMgB_14_, and Al_4_B_2_O_9_.

The experimental XRD pattern of the obtained composite is well approximated by the calculated integral intensity (Figure 2a). The refined parameters of the main phases slightly differ from the reference. According to the results obtained using the light element analyzer LECO ONH, the main source of oxygen for the formation of the Al_4_B_2_O_9_ oxide phase is boron powder (the oxygen content is 1.1 wt%).

### 3.3. Microstructure of the AlMgB_14_-TiB_2_ Composite

**Figure 3:**
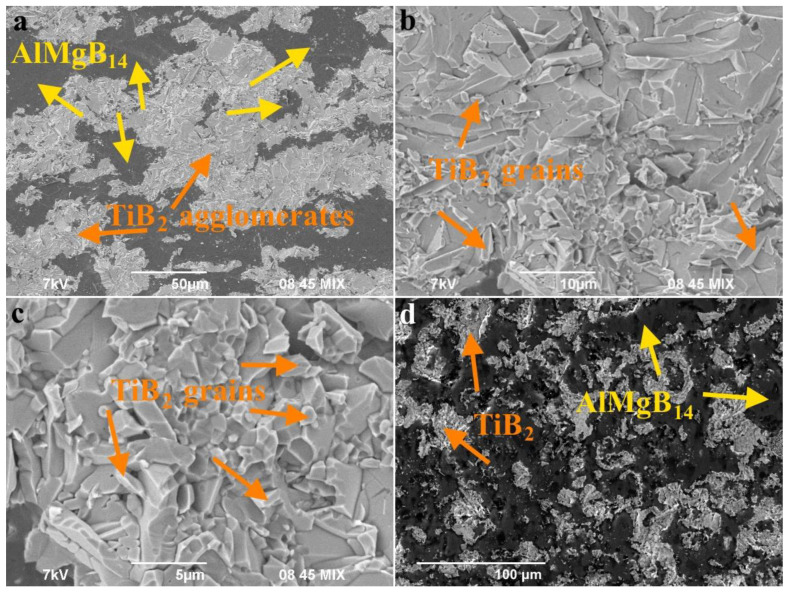
SEM images of the fracture surface (**a**–**c**) and the surface (**d**) of the AlMgB_14_-TiB_2_ composite.

The microstructure of the fracture surface and the near-surface region of the spark plasma-sintered AlMgB_14_-TiB_2_ composite are shown in Figure 3. The structure of the obtained composite is not uniform. According to the EDX results, the Ti and B elements were found in the light areas (should be TiB_2_ phase). In the dark areas, the B, Al and Mg elements were found in the ratio corresponding to the AlMgB_14_ phase. AlMgB_14_ grains have an average size of 3–5 μm, while the TiB_2_ grains have an average size of 1–3 μm. The structure of the composite also contains large agglomerates of TiB_2_, which are formed from agglomerates in the AlMgB_14_-TiB_2_ precursor [18].

### 3.4. Hardness and Density of the AlMgB_14_-TiB_2_ Composite

According to the nanohardness measurement results, in the areas corresponding mainly to AlMgB_14_, the average Vickers microhardness is 32.5 GPa. In the regions corresponding to TiB_2_, the average Vickers microhardness hardness is 33.1 GPa. In the AlMgB_14_-TiB_2_ interlayer, the average hardness of the sample is 37.4 GPa with a maximum detected hardness value of 44.1 GPa. The obtained results are similar to the results of other authors (Table 3) [4,7,19,20]. The density of the obtained sample is 3.271 g/cm^3^ at a theoretical density of 3.29 g/cm^3^ (pure 50 wt% of TiB_2_ + 50 wt% of AlMgB_14_ composite).

### 3.5. Tribological Behavior of the AlMgB_14_-TiB_2_ Composite

**Figure 4:**
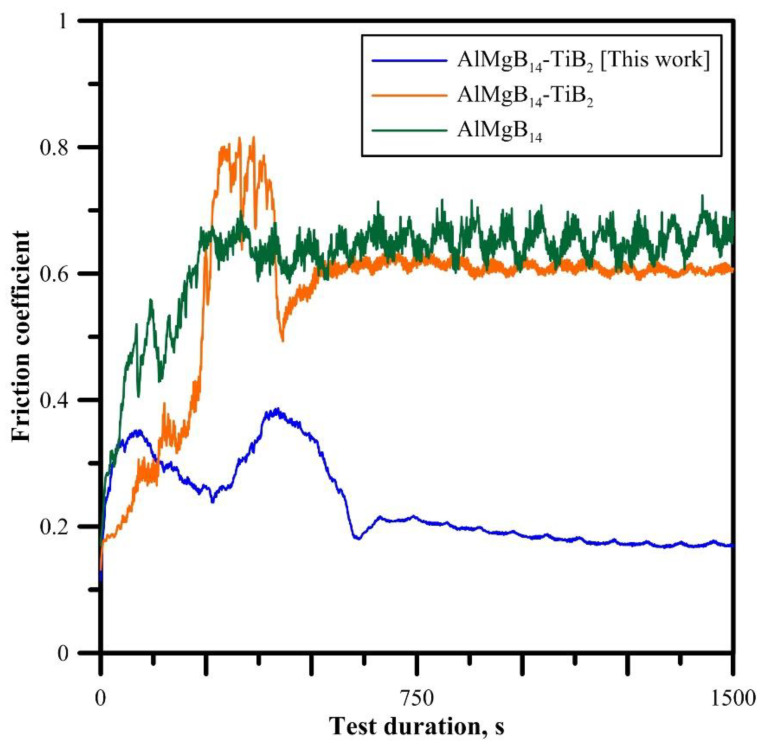
Friction coefficient of the obtained AlMgB_14_-TiB_2_ composite under dry conditions.

Figure 4 shows the curve of the change in the friction coefficient of the obtained AlMgB_14_-TiB_2_ composite. To compare this result, we also measured the friction coefficient of the AlMgB_14_ and 30 wt% TiB_2_ samples obtained in our previous works [10,23]. It was found that the friction curve of the obtained AlMgB_14_-TiB_2_ sample has a downward trend, while the curves of the AlMgB_14_ and AlMgB_14_-TiB_2_ samples, on the contrary, have an upward trend. Moreover, the change in the values of the friction coefficient of the obtained AlMgB_14_-TiB_2_ composite has an atypical character in comparison with other samples: as can be seen from Figure 4, two extrema were found in the range of the COF values of 0.35 and 0.4, respectively. After reaching the value of 0.4, the friction coefficient sharply decreases to a value of 0.18, increases to 0.22 and decreases linearly to 0.18. At the same time, according to the XRD results, in contrast to the AlMgB_14_ [23] and AlMgB_14_–30 wt% TiB_2_ [10] samples, the Al_4_B_2_O_9_ phase was found in the obtained composite. A similar result was observed in [7], where due to the plastic deformation and elevated temperature TiO_2_ particles formed a lubrication layer. Thus, it can be assumed that during friction, the friction coefficient increases until some particles (presumably, Al_4_B_2_O_9_) appear on the surface of the sample and form a lubricating coating. After that, a decrease in the friction coefficient is observed. 

## 4. Discussion

### 4.1. Formation of the Al_4_B_2_O_9_ Phase

According to the XRD results (Table 2), the Al_4_B_2_O_9_ phase was found in the obtained AlMgB_14_-TiB_2_ composite. It is known that the optimum temperature for obtaining AlMgB_14_-based materials is 1400 °C [4]. In work [24], a mechanism for the decomposition of AlMgB_14_ during spark plasma sintering was proposed. It was found that, upon the local overheating of the powder mixture (1470 °C) during spark plasma sintering, AlMgB_14_ decomposes and the decomposition products react with MgAl_2_O_4_ spinel, forming the Al_4_B_2_O_9_ borate. In this work, the overheating of the powder mixture was also observed both during the preparation of the AlMgB_14_-TiB_2_ precursor (the synthesis temperature was 1980 °C) and during the fabrication of the AlMgB_14_-TiB_2_ composite (the sintering temperature was 1500 °C). It should also be noted that the main impurity phase MgAl_2_O_4_ [4] was not detected in the AlMgB_14_-TiB_2_ composite. Thus, it is confirmed that AlMgB_14_ decomposes and reacts with MgAl_2_O_4_, forming aluminum borate Al_4_B_2_O_9_, during SHS and SPS due to local overheating.

### 4.2. Structure and Phase Composition of the AlMgB_14_-TiB_2_ Composite

The crystalline phases of TiB_2_ and AlMgB_14_ are precipitated in the composite material. Indeed, quantitative phase analysis (Table 2, Figure 2b) showed that the contribution of the intensity of the TiB_2_ lattice (Figure 2b, mark 4) is dominant in the crystalline part of the integrated intensity (Figure 2a, mark 2). For these phases, complete structural information was found (Table 2). An increase in the lattice parameters was found, but the volume of the AlMgB_14_ crystal lattice decreased relative to the reference, which is associated with a change in the shape of the lattice.

Complete information about the structure makes it possible to elucidate from the first principles the stability of the lattices of the main phases of the AlMgB_14_-TiB_2_ composite. The details of the calculations are given in Section 4.3. The estimates from the first principles showed that the lattice energies of the main phases (TiB_2_, AlMgB_14_ and Al_4_B_2_O_9_) are negative; therefore, they are stable (Table 2). The Al_4_B_2_O_9_ supercell is also stable to the lattice separation of the Al_2_O_3_ and B_2_O_3_ phases (Section 4.3). Indeed, its binding energy is |ΔEst| ≈ 2163 eV. The analysis of the morphology of the fracture surface (Figure 3a–c) showed that TiB_2_ agglomerates (light areas) are characterized by a clear separation of interfaces between crystallites of various shapes and sizes with an arbitrary direction, while AlMgB_14_ (dark areas), on the contrary, are characterized by a blurred surface relief (Figure 3c).

### 4.3. Mechanism of Self-Lubricity of the AlMgB_14_-TiB_2_ Composite

An analysis of the literature data [7,8,9] shows that the nature of the change in the friction coefficient under the dry conditions of the obtained AlMgB_14_-TiB_2_ composite is not typical for AlMgB_14_ materials and AlMgB_14_-TiB_2_ composite materials (Figure 4). Thus, of particular interest are studies aimed at identifying the mechanism for reducing the friction coefficient in the obtained composite. Since the Al_4_B_2_O_9_ lattice is a supercell with the chemical formula {2(Al_2_O_3_) × (B_2_O_3_)} (that is, it contains B_2_O_3_ particles in its compound, which are responsible for the lubrication of AlMgB_14_-TiB_2_ composite materials [2]), it is assumed that the dry friction process is significantly influenced by B_2_O_3_ particles, as well as the morphology of AlMgB_14_. 

**Figure 5:**
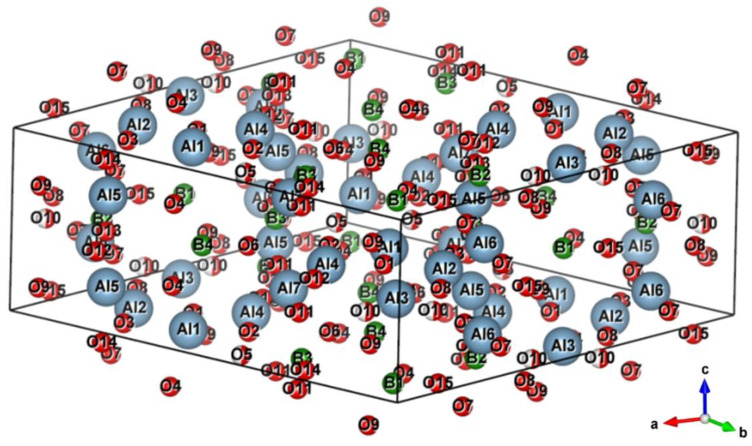
3D image of the Al_4_B_2_O_9_ lattice after refinement of structural parameters by the Rietveld method.

To reveal the mechanism of self-lubrication of the obtained AlMgB_14_-TiB_2_ composite, the stability of the Al_4_B_2_O_9_ phase was calculated. Since Al_4_B_2_O_9_ is a supercell with the chemical formula {2(Al_2_O_3_) × (B_2_O_3_)} (Figure 5), its stability ΔEst can be estimated by Formula (1):

Δ*E_st_* = Δ*E_Al_4_B_2_O_9__* − (*x*Δ*E_Al_2_O_3__* + *y*Δ*E_B_2_O_3__*) (1)

where Δ*Est* < 0 and x and y are the relative contents of the Al_2_O_3_ and B_2_O_3_ phases in the Al_4_B_2_O_9_ supercell. Δ*E_Al_4_B_2_O_9__* is the supercell energy. The lattice energies of the Al_2_O_3_ and B_2_O_3_ phases were calculated from the first principles and were Δ*E_Al_2_O_3__* = −8590.47 eV and Δ*E_B2O3_* = −4243.67 eV. The lattice energy of Al_4_B_2_O_9_ is given in Table 2, and the coefficients are: x = 16/6, y = 8/3. The estimates showed that Δ*Est* ≈ −2163 eV, demonstrating that the Al_4_B_2_O_9_ lattice is substantially stable. 

In the next stage, quantum-chemical calculations of the elastic moduli of the main phases were performed using the method [25,26]. The results are given in Table 4. The obtained results are in a good agreement with the experimentally observed value. The elastic lattice moduli of Al_4_B_2_O_9_ are significantly smaller than those of TiB_2_ and AlMgB_14_. It should also be noted that the elastic moduli of the AlMgB_14_ are less than the corresponding values of TiB_2_; AlMgB_14_ can be a “viscous” base of the composite during the dry friction. Thus, it is assumed that the Al_4_B_2_O_9_ particles are squeezed out of the rigid TiB_2_ matrix and viscous AlMgB_14_ matrix onto the composite surface and lubricate it, which leads to a decrease in the friction coefficient.

**Figure 6:**
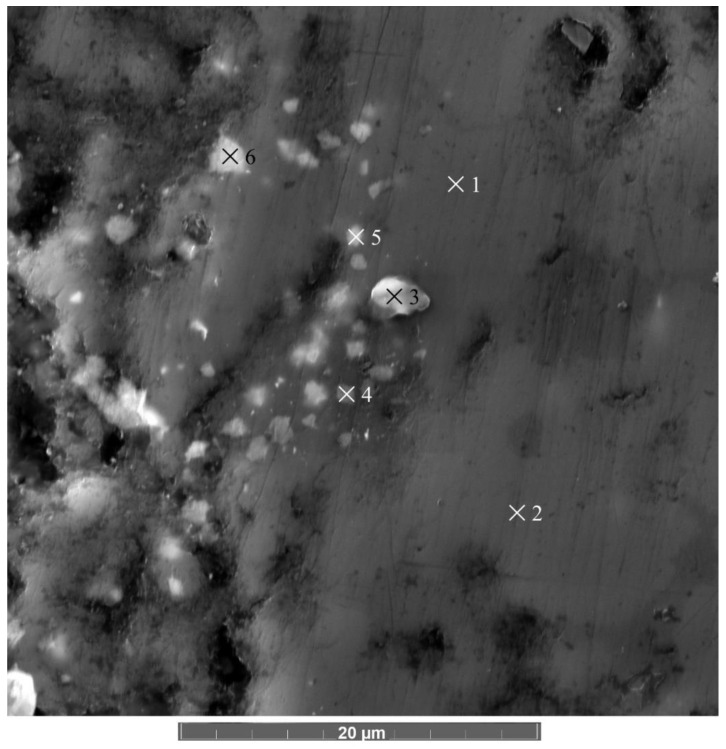
SEM image of the relief of the AlMgB_14_-TiB_2_ composite after friction testing (on the surface, the areas where the elemental composition is determined are marked).

To find particles of the Al_4_B_2_O_9_ composition on the surface and thus prove this hypothesis, the elemental composition of the EDX method was determined in the track region. Figure 6 shows a fragment of the topographic surface of the AlMgB_14_-TiB_2_ composite in the track region after measuring the friction coefficient.

The mass fraction of the elements in the corresponding regions is given in Table 5. The results of the estimates of the molar fraction indicate that in spectra 3, 4, 5 and 6 (Figure 6), the areas of destruction of the Al_4_B_2_O_9_ particles as a result of the sliding friction with the release of the B_2_O_3_ and Al_2_O_3_ compounds of a variable composition are found.

Thus, Al_4_B_2_O_9_ particles are distributed on the surface of a sample consisting of a rigid TiB_2_ matrix with a “viscous” base of the AlMgB_14_ (spectra 1 and 2) phase.

As previously reported in the Introduction, the effect of squeezing out particles onto the surface of AlMgB_14_-based materials was observed in other studies. A comparison of the friction coefficients is shown in Table 6. In [27,28], it was found that in the Al_2_O_3_-TiB_2_ composites, during friction at elevated temperatures in the air, an oxide layer is formed, which lubricates the sample. In turn, the formation of the oxide layer is due to the plastic deformation of the tribo-contact region and the fact that the critical shear modulus of the oxide layer is much less than the shear modulus of the substrate. In this work, similar results were obtained: a sharp decrease in the COF is associated with the release of B_2_O_3_ and Al_2_O_3_ particles of a variable composition from the rigid TiB_2_ matrix with the “viscous” base of the AlMgB_14_ phase onto the composite surface during friction. These particles form a lubricating tribolayer and reduce the friction coefficient. In other words, the effect of the self-lubrication of the surface of the composite material AlMgB_14_-TiB_2_ with Al_4_B_2_O_9_ particles is observed. 

Thus, the Al_4_B_2_O_9_ additive can be used as a lubricant in AlMgB_14_-TiB_2_ composite materials under dry friction conditions. It should be noted that according to the presented results, Al_4_B_2_O_9_ does not negatively affect the hardness of the AlMgB_14_-TiB_2_ composite system. In the future, of particular interest is the study of AlMgB_14_-TiB_2_ composite materials that use prereacted Al_4_B_2_O_9_ as the initial precursor.

## 5. Conclusions

1. The AlMgB_14_-TiB_2_ composite was spark plasma sintered from the SHS precursor.
2. The main phases in the obtained composite are TiB_2_, AlMgB_14_ and Al_4_B_2_O_9_.
3. The maximum hardness of the composite is 44.1 GPa at a density of 3.271 g/cm^3^.
4. The presence of oxygen in the form of the Al_4_B_2_O_9_ phase led to unexpectable results: the friction coefficient of the obtained AlMgB_14_-TiB_2_ composite in dry conditions against the Al_2_O_3_ counter-specimen is 0.18. It is approximately four times lower than the friction coefficient of pure ceramic AlMgB_14_ (COF, 0.7).
5. It was found that Al_4_B_2_O_9_ particles are responsible for the low friction coefficient: the elastic moduli of Al_4_B_2_O_9_ are significantly smaller than the elastic moduli of AlMgB_14_ and TiB_2_. Thus, during the friction, due to the plastic deformation of the tribo-contact region, Al_4_B_2_O_9_ particles are squeezed out onto the composite surface, form the lubricating layer and reduce the friction coefficient.

## Figures and Tables

**Table 1:** Characteristics of the raw powders.

Raw Precursors	Particle Size, μm	Purity, %
Ti	140	≥99.2
Amorphous B	0.6	≥98.7
Al_12_Mg_17_	15	≥99.2

**Table 2:** Structural parameters of the detected phases.

Phase	State	a, Å	b, Å	c, Å	α = β	γ	V, Å^3^	E, eV
TiB_2_	Reference	3.009	3.009	3.262	90.00	120.00	25.578	−210.90
	Refined	3.033	3.033	3.240	90.00	120.00	25.816	−210.89
AlMgB_14_	Reference	5.848	8.115	10.313	90.00	90.00	489.419	−7149.38
	Refined	5.690	8.050	10.357	90.00	90.00	474.448	−7148.67
Al_4_B_2_O_9_	Reference	14.792	15.028	5.534	90.00	90.96	1230.15	−34471.7
	Refined	14.460	15.036	5.475	90.00	85.51	1152.61	−34469.3

**Table 3:** Comparison of hardness values of AlMgB_14_-based materials.

Composition	Hardness Value, GPa	Reference
AlMgB_14_ + 50 wt% TiB_2_	40–46	[4]
AlMgB_14_ + 30 wt% Si	27	[8]
AlMgB_14_-5Al	22.4	[21]
AlMgB_14_	26.7	[22]
AlMgB_14_ + 50 wt% TiB_2_	37.4	This work

**Table 4:** Elastic moduli (GPa) of the main phases of the obtained material.

Phase	Young’s Modulus	Young’s Modulus (Experimental Value) *	Bulk Modulus	Shear Modulus
TiB_2_	491.8	480.1	245.9	210.8
AlMgB_14_	424.7		237.3	182.9
Al_4_B_2_O_9_	206.2		132.0	83.2

*—From nanohardness measurements.

**Table 5:** Spectra of elements in selected places on the surface of the AlMgB_14_-TiB_2_ composite.

Element	Mark 1	Mark 2	Mark 3	Mark 4	Mark 5	Mark 6
	wt%					
B	84.4	93.1	66.7	49.0	55.8	43.9
Al	8.8	8.5	8.7	17.7	15.0	20.9
Mg	6.1	6.0	5.9	9.3	7.5	9.7
Ti	0.4	2.1	0.4	0.4	0.3	0.3
O	−	0.2	18.3	23.5	21.3	25.1
Estimated phase	AlMgB_14_	AlMgB_14_/TiB_2_	Al_4_B_2_O_9_	Al_4_B_2_O_9_	Al_4_B_2_O_9_	Al_4_B_2_O_9_

**Table 6:** Comparison of friction coefficients in dry conditions of the AlMgB_14_-based materials.

Composition	Friction Coefficient	Reason of Low COF	Reference
AlMgB_14_	0.38	−	[11]
AlMgB_14_-TiB_2_	0.45−0.65	−	[7]
AlMgB_14_-TiB_2_	0.12 (800 °C)	TiO_2_ particles	[7]
AlMgB_14_-Si	0.19−0.28	316L counter-specimen Si particles	[8]
AlMgB_14_-Cu	0.2	AlMgB_14_ particles	[9]
AlMgB_14_-TiB_2_	0.18	Al_4_B_2_O_9_ particles	This work

## Data Availability

The data presented in this study are available in the article.

## Supplementary Materials

The following supporting information can be downloaded at: https://www.mdpi.com/article/10.3390/ma15238450/s1. Figure S1: Heat pattern of the SHS process. Figure S2: EDX_results to Figure 1. Figure S3: Nanohardness indentations.
