# Supplementary material for: Experimental and Theoretical Study of Ultra-Hard AlMgB14-TiB2 Composites: Structure, Hardness and Self-Lubricity
Source: Materials (Basel). 2022 Nov 27;15(23):8450. doi: 10.3390/ma15238450 (PMC9739838; doi:10.3390/ma15238450)
Supplement: Supplementary file 1 [file materials-15-08450-s001.zip › materials-2005793-supplementary.pdf]

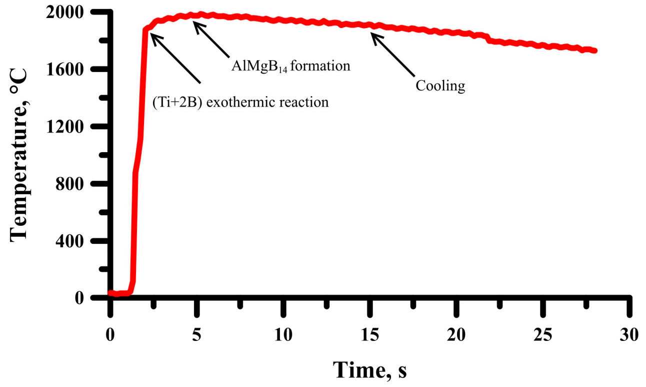

Figure S1. Heat pattern of the SHS process.

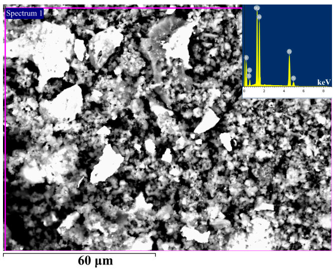

Figure S2. EDX_results to Figure 1.

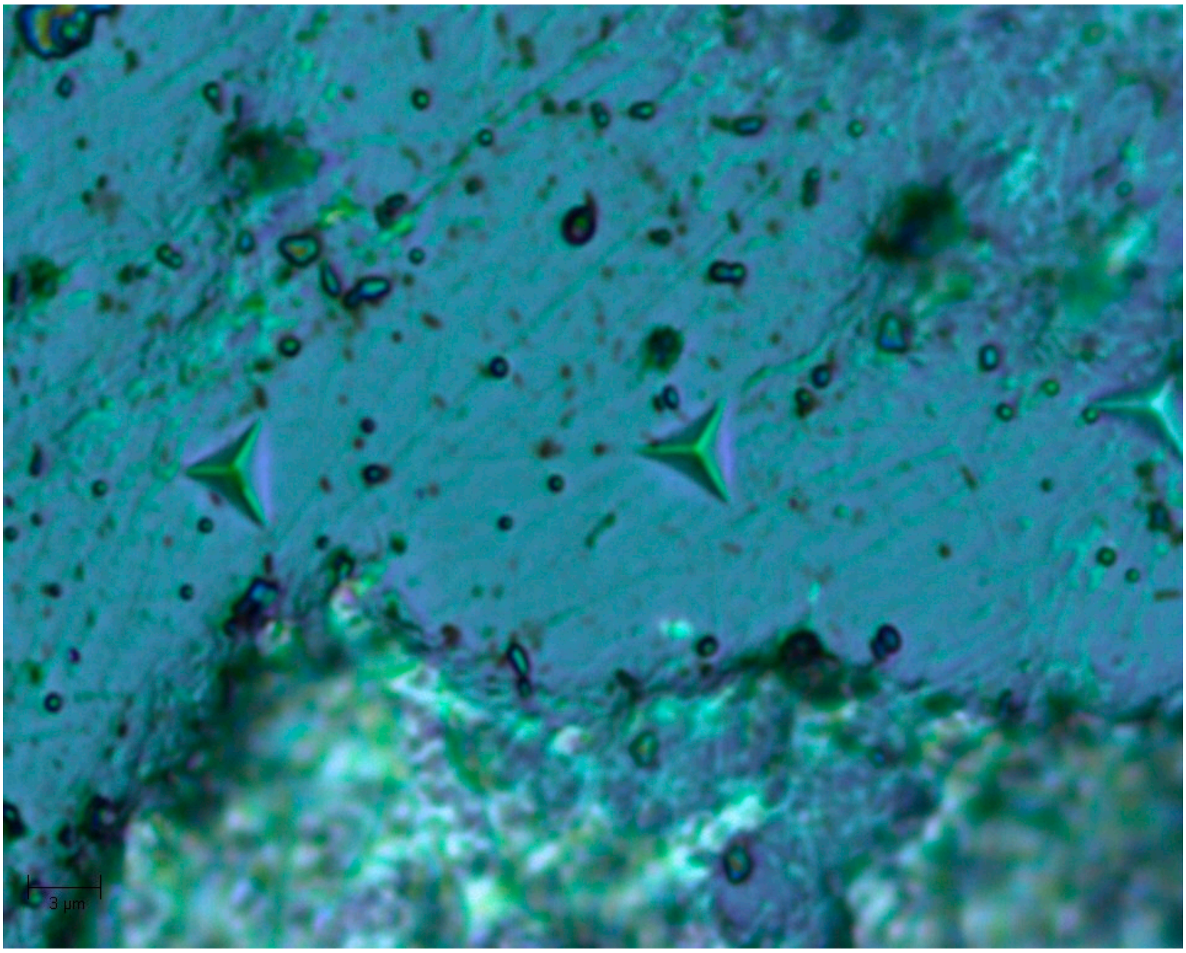

Figure S3. Nanohardness indentations.
